# The Junction of Allergic Inflammation and Atherosclerosis: Pathways and Clinical Implications—A Review

**DOI:** 10.3390/life15060964

**Published:** 2025-06-16

**Authors:** Mihaela Valcovici, Mihai Sorin Iacob, Abhinav Sharma, Ana Maria Pah, Lucretia Marin-Bancila, Marcel Mihai Vaduva Berceanu, Milan Daniel Velimirovici, Anca-Raluca Dinu, Simona Ruxanda Drăgan, Nilima Rajpal Kundnani

**Affiliations:** 1 Department VI—Cardiology, “Victor Babeş” University of Medicine and Pharmacy, 300041 Timisoara, Romaniaknilima@umft.ro (N.R.K.); 2 Doctoral School, “Victor Babeş” University of Medicine and Pharmacy, 300041 Timisoara, Romania; 3 Faculty of Medicine, “Victor Babeş” University of Medicine and Pharmacy, 300041 Timisoara, Romania; 4 Department XVI—Orthopedics-Traumatology—I, Urology and Medical Imagistics, “Victor Babeş” University of Medicine and Pharmacy, 300041 Timisoara, Romania; 5 Department I—Nursing, University Clinic of Practical Abilities, “Victor Babeş” University of Medicine and Pharmacy, 300041 Timisoara, Romania; 6 Department XVI, Medical Recovery, “Victor Babeş” University of Medicine and Pharmacy, 300041 Timisoara, Romania; 7 Research Center for Assessment of Human Motion and Functionality and Disability, “Victor Babeş” University of Medicine and Pharmacy, Eftimie Murgu Square, No. 2, 300041 Timisoara, Romania; 8 Pius Brinzeu Emergency Clinical County Hospital, Bld Liviu Rebreanu, No. 156, 300723 Timisoara, Romania; 9 Research Centre of Timisoara Institute of Cardiovascular Diseases, “Victor Babeş” University of Medicine and Pharmacy, 300041 Timisoara, Romania

**Keywords:** allergy, inflammation, t cells, mastocytes, atherosclerosis, cardiovascular disease

## Abstract

**Background**: Cardiovascular disorders, especially atherosclerosis, have been associated with allergic inflammation. In addition to traditional inflammatory responses, there is evidence that the development and instability of coronary artery plaque may be influenced by effector cells of allergic inflammation. This review examines the phases of allergic pathology, the immunological mechanisms of atherosclerosis, and the clinical link between allergic diseases (asthma, atopic dermatitis, allergic rhinitis, and food allergy) and cardiovascular disease (CVD), along with future therapeutic perspectives. **Material and Method**: A literature search was conducted in PubMed, Google scholar; ScienceDirect, Scopus, and studies published between 2014–2024 were taken into consideration. Keywords included allergic inflammation, eosinophils, mast cells, reactive oxygen species, atherosclerosis, Th2 cells, and cytokines. Epidemiological studies and review articles were included. **Results**: Emerging evidence suggests that allergic inflammation contributes to atherosclerosis through interconnected mechanisms such as eosinophil activation, reactive oxygen species production, mast cell degranulation, and endothelial dysfunction. Th2-driven immune responses, which are mediated by cytokines such as IL-4, IL-5, and IL-13, as well as eosinophil activity and mast cell degranulation, play a crucial role in vascular inflammation and plaque progression. Additionally, changes in lipid metabolism contribute to this process. Epidemiological studies support this connection, indicating that patients with chronic allergic conditions such as asthma, allergic rhinitis, food allergy, and atopic dermatitis experience increased cardiovascular morbidity. However, most current data are observational, and our understanding of the underlying mechanisms in humans remains limited, often relying on insights gained from preclinical models. **Conclusions**: A potential mechanism for cardiovascular risk is suggested by the interaction between atherosclerosis and allergic inflammation. Promising alternatives for treating allergic inflammation and cardiovascular issues include novel treatments like cytokine inhibitors, mast cell stabilizers, and biologics that target certain pathways. Further research is necessary to see whether concentrating on allergy pathways could lead to innovative treatments for cardiovascular disorders or vice versa.

## 1. Introduction

### 1.1. Overview of Atherosclerosis and Its Risk Factors

Atherosclerosis is a condition characterized by the narrowing and hardening of arteries due to the accumulation of plaque on the arterial walls. This plaque is composed of fatty deposits, cholesterol, calcium, and other blood-borne substances, leading to restricted blood flow and various associated health complications such as heart attacks, strokes, and peripheral artery disease [1]. The progression of atherosclerosis is often gradual and can be influenced by factors including high blood pressure, dyslipidemia, smoking, and diabetes. Low-density lipoproteins (LDLs) are particularly instrumental in the initiation and advancement of atherosclerosis [2]. Through interactions with extracellular structures, such as arterial wall proteoglycans, LDLs become sequestered in the subendothelial space, where they may undergo oxidation (oxLDL) or other modifications [3]. This sequestration triggers an autoimmune response to LDL and other antigens, which can either exacerbate or ameliorate the course of the disease. Oxidized LDL is perceived as harmful by the immune system. White blood cells called macrophages are recruited to the site of inflammation to engulf and digest the oxidized LDL [1]. This process is intended to remove the harmful substance, but can lead to further issues. Macrophages that take up oxidized LDL can become overloaded with lipids and transform into foam cells. Foam cells are a hallmark of early atherosclerotic lesions [4]. While they attempt to clear the oxidized LDL, they contribute to the accumulation of lipid deposits in the arterial wall. As foam cells die, they release their contents, which include lipids and inflammatory mediators [5]. This process contributes to the formation of a fatty streak, an early stage of atherosclerotic plaque. Over time, additional lipids, cells, and connective tissue (including collagen) accumulate, forming a more complex plaque. The plaque consists of a lipid core and a fibrous cap [6]. Smooth muscle cells migrate to the site and proliferate, producing extracellular matrix components such as collagen and elastin. The plaque continues to grow as more lipids and cells accumulate. It can become quite large and harden, narrowing the artery and restricting blood flow [1]. The fibrous cap can also become thin and vulnerable. If the cap ruptures, it exposes the underlying lipid core to the bloodstream, which can trigger the formation of a blood clot (thrombus). This clot can further block the artery or break off and travel to other parts of the body, potentially causing a heart attack or stroke [7].

### 1.2. Introduction to Allergic Inflammation

When an individual with a genetic predisposition to allergies is initially exposed to minimal levels of allergens, specialized antigen-presenting cells (APCs) internalize the allergen, leading to its intracellular digestion into peptide fragments. These peptides are subsequently presented in an HLA class II groove on the surface of the APCs [8]. Dendritic cells are the most “professional” of the antigen-presenting cells (APCs); they migrate to lymph nodes, and prime and activate naive T cells to differentiate, proliferate, and clonally expand into various immune cell subsets, such as Th1, Th2, Th17, Tc1, Tc2, Tc17, and regulatory T cells [9], as well as the recently identified follicular T helper cells 13 (Tfh13) [10] that produce interleukin-4 (IL-4), interleukin-13 (IL-13), and IL-21. These Th2 cytokines play a pivotal role in stimulating B cells to undergo class switching and express immunoglobulin E (IgE), exemplified by interleukin-13 (IL-13) and interleukin-4 (IL-4). Furthermore, the Th2 cytokines instigate eosinophil proliferation in the bone marrow, typified by interleukin-5 (IL-5), and upregulate adhesion molecules on blood vessels to facilitate the extravasation of circulating inflammatory cells involved in allergic inflammation, such as eosinophils and basophils [11]. IgE specific to the allergen, generated during the initial exposure, binds to IgE receptors on mast cells and basophils with a high-affinity receptor (FcεRI). Upon subsequent exposure to the specific allergen, these IgE-sensitized mast cells release preformed pro-inflammatory and vasoactive mediators (histamine, tryptase, chymase, etc.), or synthesized de novo (prostaglandins, leukotrienes, PAF, etc.) [12], thereby contributing to the allergic inflammatory response. This mediator release results in hallmark symptoms of allergic reactions, including pruritus, edema, and increased mucus production [8].

**Figure 1:**
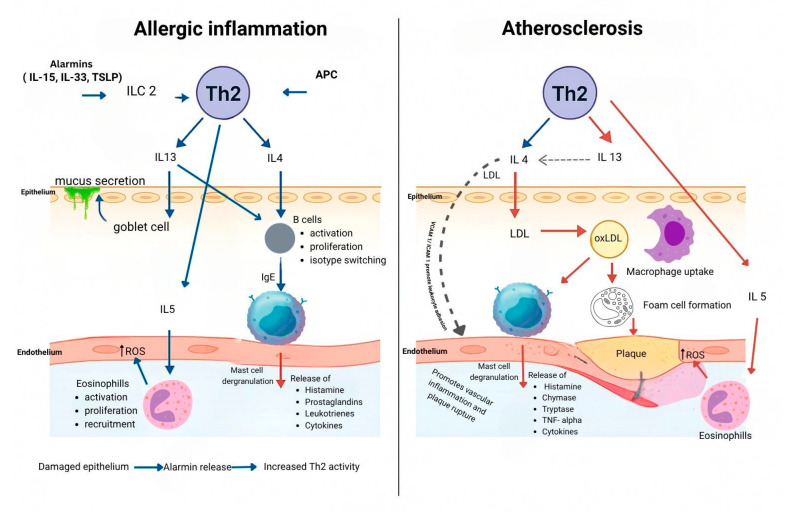
Molecular mechanisms of allergic inflammation and atherosclerosis.

Innate lymphoid cells type 2 (ILC2) are a group of immune cells that play a crucial role in type 2 immune responses, which are important for allergic inflammation, tissue repair, and defense against parasites. Unlike T cells, ILC2 does not have antigen-specific receptors. Instead, they are activated by cytokines known as alarmins, which include IL-25, IL-33, and thymic stromal lymphopoietin (TSLP) [13]. These alarmins are released by epithelial cells in reaction to allergens, infections, or tissue damage, serving as danger signals to trigger immune responses. When activated, ILC2 produces type 2 cytokines such as IL-4, IL-5, and IL-13. These cytokines are responsible for the characteristic features of allergic diseases like asthma and atopic dermatitis [14]. Specifically, IL-4 and IL-13 promote mucus production and airway hyperreactivity [15], while IL-5 recruits eosinophils [16], which further amplify inflammation. Alarmins trigger the allergic cascade by activating ILC2, which in turn enhances the immune response (Figure 1).

This review explores the various phases of allergic pathology and the fundamental immunological mechanisms involved in atherosclerosis. It focuses on cellular mechanisms and the clinical links between allergic diseases—specifically asthma, atopic dermatitis, allergic rhinitis, and food allergies—and cardiovascular disease (CVD). Additionally, the review discusses future perspectives and potential therapeutic strategies for managing the intersection of allergic diseases and CVD.

## 2. Materials and Methods

We conducted a comprehensive search of four electronic databases, PubMed, Google Scholar, ScienceDirect, and Scopus, focusing on studies published between January 2014 and January 2024. Our search strategy combined several keywords and MeSH terms, including “allergic inflammation”, “eosinophils”, “mast cells”, “reactive oxygen species”, “atherosclerosis”, “Th2 cells”, and “cytokines”, using Boolean operators (AND/OR) to refine the results. We included only peer-reviewed human studies, epidemiological and cohort studies, English publications, and relevant review articles that explored the link between allergic inflammation and cardiovascular diseases, particularly atherosclerosis. We excluded case reports, animal studies, publications in languages other than English, and studies without full-text access. Two reviewers independently screened the titles and abstracts of the identified studies, followed by full-text assessments to determine which studies met our eligibility criteria. Any disagreements were resolved through consensus. We extracted data related to inflammatory pathways, cellular mediators, and clinical correlations. Due to the varying study designs, we performed a qualitative synthesis instead of a meta-analysis.

### Linking Allergies to Atherosclerosis (and Cardiovascular Disease)

Recent studies suggest that allergic inflammation may significantly contribute to the initiation and progression of atherosclerosis. This theory is based on the shared key inflammatory pathways and immune responses observed in both conditions [17,18]. Specifically, the Th2-mediated immune response commonly seen in allergic inflammation, characterized by elevated levels of cytokines such as interleukin (IL)-4, IL-5, and IL-13, as well as increased activity of eosinophils and mast cells, may play a role in the chronic vascular inflammation seen in atherosclerosis [19,20]. These shared mechanisms imply that allergic inflammation could worsen endothelial dysfunction, promote the infiltration of immune cells into the arterial wall, and accelerate the formation of atherosclerotic plaques, ultimately increasing the risk of cardiovascular events (Table 1). Given the global burden of cardiovascular diseases, exploring the role of allergic inflammation in atherosclerosis is very relevant. This subject relates to the comprehensive understanding of chronic inflammation and cardiovascular risk.

## 3. Pathophysiology and Immune Cell Infiltration and Activation

### 3.1. Th2 Cells and Cytokines and Chemokines

Cytokines involved in allergic inflammation, such as interleukin-4 (IL-4) and interleukin-13 (IL-13), play significant roles in driving the immune responses characteristic of allergic diseases [11]. There is emerging evidence that Th2 cell-driven immune responses contribute to the chronic inflammatory environment found in atherosclerotic plaques through several mechanisms. While atherosclerosis has traditionally been associated with Th1-driven inflammation [27], emerging research indicates that Th2 cells also play a significant role in this chronic inflammatory state. Th2 cells are characterized by their production of cytokines such as IL-4, IL-5, and IL-13, which are central to shaping the immune landscape of atherosclerotic plaques [28]. IL-4 promotes the expression of adhesion molecules on endothelial cells, enhancing the recruitment of monocytes and other immune cells to the arterial wall [29]. In contrast, IL-13 has been reported as atheroprotective [30]. Additionally, these cytokines reinforce the recruitment of more Th2 cells and other immune cells to the endothelium while promoting the differentiation of monocytes into macrophages. These macrophages can take up oxidized LDL, leading to the formation of foam cells, which are key contributors to plaque development [31]. IL-5, another cytokine produced by Th2 cells, facilitates the recruitment and activation of eosinophils. Eosinophils release granule proteins and reactive oxygen species (ROS) that can damage endothelial cells and increase oxidative stress within plaques [32]. While human plasma IL-5 levels do not predict cardiovascular disease risk, elevated IL-5 levels have been linked to reduced carotid intima-media thickness and increased production of oxidized LDL-specific antibodies [33].

### 3.2. Mast Cells

Mast cells, which play a crucial role in allergic reactions, are increasingly recognized for their involvement in cardiovascular diseases, particularly atherosclerosis. These immune cells can infiltrate the arterial walls and contribute significantly to the formation of atherosclerotic plaques. Within the tissue environment, mast cells, in conjunction with eosinophils, release a variety of cytokines (e.g., TNF-α, IL-6), growth factors (e.g., VEGF), proteases (e.g., tryptase, chymase), and vasoactive substances [34]. These mediators are integral to the processes of tissue inflammation and remodeling, exacerbating vascular inflammation, which in turn plays a pivotal role in the progression of atherosclerosis. The release of these inflammatory mediators leads to several key events: they enhance the permeability of blood vessels, promote the adhesion of leukocytes (white blood cells) to the endothelial cells lining the blood vessels, and activate these endothelial cells themselves. This chain reaction cultivates an inflammatory environment, further leading to the progression of atherosclerosis [5]. Additionally, the activated mast cells secrete histamine and leukotrienes, both of which are powerful inflammatory molecules. Histamine, in particular, increases vascular permeability and contributes to the activation of the endothelial cells, facilitating a process that allows low-density lipoprotein (LDL) particles and inflammatory cells to infiltrate the arterial intima, a critical step in plaque formation [35]. Mast cells are frequently located near the microvessels that develop within the plaques, where they release angiogenic factors such as vascular endothelial growth factor (VEGF) and basic fibroblast growth factor (bFGF) [36]. These angiogenic molecules stimulate the growth of new blood vessels, which can further complicate the atherosclerotic process. One of the most well-studied pathways for mast cell activation is the IgE-dependent mechanism. In this pathway, allergens induce cross-linking of IgE antibodies that are bound to the Fcε receptors on the surfaces of mast cells [37]. This activation cascade is prominently linked to the manifestation of allergic responses. Research shows that patients with elevated IgE levels face a higher risk of cardiovascular mortality. This risk is particularly significant in individuals without baseline cardiovascular diseases compared to those who already have cardiovascular conditions [38], suggesting a potential role for mast cell activity in the development of cardiovascular mortality. Additionally, coronary vasospasm is another significant factor that contributes to the development of atherosclerosis [39]. Histamine released from activated mast cells can trigger coronary vasoconstriction via histamine 2 receptors (H2Rs), potentially leading to serious events such as myocardial infarction (heart attack) [40].

**Figure 2:**
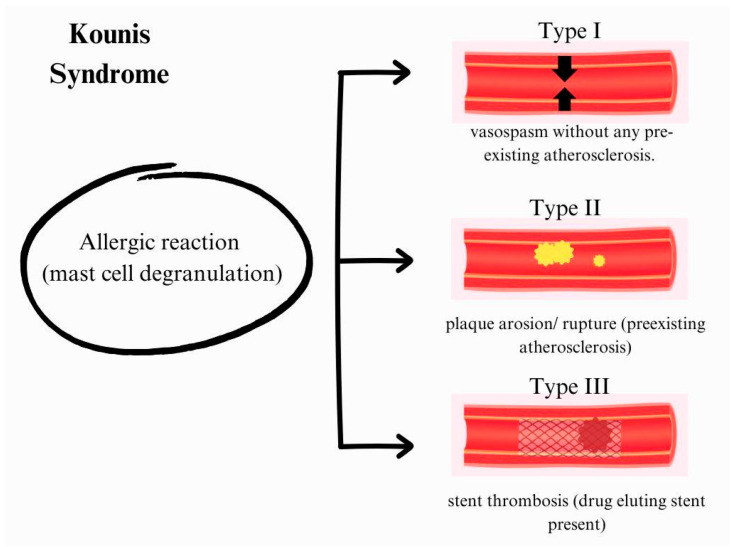
Schematic representation of the three types of Kounis syndrome.

The connection between allergies and acute coronary events is illustrated by Kounis syndrome [41]. This syndrome can manifest as chest pain or myocardial infarction triggered by an allergic, hypersensitivity, or anaphylactic reaction. The primary underlying mechanism involves mast cell degranulation, during which inflammatory mediators are released due to an antigen–antibody reaction occurring on the surface of mast cells and basophils. Kounis syndrome is classified into three types (Figure 2) [42]. Type I occurs in patients with normal or nearly normal coronary arteries. In this case, the allergic reaction causes coronary artery spasm (vasospasm) without any pre-existing atherosclerosis. Type II consists of patients having an allergic reaction that can lead to plaque rupture or erosion because they have an existing coronary artery disease, such as atherosclerosis. In type III, the release of inflammatory mediators in patients with coronary artery stent thrombosis (who have previously undergone percutaneous coronary intervention (PCI) and have drug-eluting stents) can trigger stent thrombosis [42,43].

Through these interconnected pathways, mast cells are shown to be active participants not only in allergic responses but also in the complex processes that underlie atherosclerosis and its associated cardiovascular risks.

### 3.3. Eosinophils

Eosinophils, which are often elevated in allergic conditions such as asthma and atopic dermatitis [34], are also recruited to the vascular wall in cases of atherosclerosis [5]. Interestingly, eosinophils are absent in stable human plaques but are found in ruptured plaques [44]. They release toxic granule proteins, like eosinophil cationic protein (ECP), and pro-inflammatory cytokines that facilitate the formation of atherosclerotic plaques by enhancing the exposure of von Willebrand factor on endothelial cells, which increases platelet adhesion [45]. ECP represents a marker of eosinophil activity, and increased serum ECP levels are related to the presence and severity of asthma and other allergic diseases [46]. During arterial thrombosis, eosinophils are rapidly recruited in an integrin-dependent manner and interact with platelets, activating eosinophils. The interactions between platelets and eosinophils contribute to reciprocal activation and promote plaque formation. Activated eosinophils release granules or extracellular traps (EETs) containing components like ECP [47], which induces the expression of CD54 (ICAM-1) on endothelial cells, facilitating and promoting inflammation [48]. Moreover, CCL11 (eotaxin-1), a potent eosinophil chemoattractant and activator, is overexpressed in atherosclerotic lesions [45]. These findings suggest that activated eosinophils may play a significant role in atherosclerosis, potentially through the activation and release of their cytotoxic granules.

Eosinophils are a major source of reactive oxygen species (ROS) in conditions like asthma and other allergic diseases. In the context of asthma, activated eosinophils release ROS such as superoxide anions, hydrogen peroxide, and other oxidative molecules as part of the inflammatory response [32]. Several studies have consistently demonstrated that ROS accumulation and oxidative stress drive atherogenesis: reactive oxygen species (ROS) damage endothelial cells, reduce nitric oxide (NO) availability, and impair vasodilation, leading to early atherosclerosis [49]. They oxidize LDL, forming oxLDL, which macrophages take up, resulting in foam cell formation and plaque development [50]. ROS also promotes smooth muscle cell proliferation, thickening the arterial wall, and activating matrix metalloproteinases (MMPs), degrading the extracellular matrix and destabilizing plaques, increasing the risk of rupture and thrombosis [51].

### 3.4. Lipid Metabolism

Recent studies examining individuals diagnosed with allergies reveal that allergic disease may cause metabolic changes [52,53]. Additionally, the mediators of inflammation and allergy show a correlation with lipid levels, supporting the hypothesis that inflammation, allergies, and lipid profiles may be interconnected components of the same system [52,54]. Various studies have investigated the relationship between dyslipidemia and conditions such as allergic rhinitis [55], food allergies [53], atopic dermatitis [56], and asthma [57], consistently reporting that the risk of dyslipidemia is statistically higher among individuals with these conditions. The relationship between dyslipidemia and atopic sensitization can be understood through the role of lipoproteins in the development of allergic reactions. Specifically, dyslipidemia promotes a shift toward a Th2-oriented immune response, thereby intensifying allergic inflammation [58]. Additionally, total serum cholesterol levels (including both HDL-C and LDL-C) may enhance eosinophilic inflammation in individuals with a genetic predisposition to atopy [55]. There is a significant correlation between serum cholesterol levels and increased inflammatory markers in these individuals.

Lipoproteins are crucial for immunity, with HDL being notably associated with cardiovascular diseases. However, evidence also indicates HDL’s significant role in physiological immune function [59]. Recent research has focused on the composition, distribution, and function of HDL particles in allergic and skin diseases. Studies have shown that HDL’s composition and functionality are altered in these conditions.

High-density lipoprotein (HDL) isolated from patients with allergic rhinitis shows a significantly reduced content of apolipoprotein A-I (apoA-I) and phosphatidylcholine, while having increased levels of apolipoprotein A-II (apoA-II), lysophosphatidylcholine, and triglycerides, compared to HDL from non-allergic healthy controls [55]. In patients with asthma and dermatitis, HDL often exhibits lower levels of anti-inflammatory molecules, such as apolipoproteins (e.g., apoA-I and apoA-IV) and certain lysophospholipids, which normally help suppress inflammatory responses [60,61]. Additionally, a study conducted in the United States found that serum HDL cholesterol (HDL-C) levels are independently and inversely related to blood eosinophil counts among asthmatic adults, suggesting a connection between serum HDL-C and the immune status of these individuals [61].

Notably, apolipoproteins such as ApoA-I (the primary protein component of HDL), ApoA-IV, and specific HDL-associated lysophospholipids act as endogenous anti-inflammatory mediators that potently suppress effector cell functions in eosinophils and neutrophils, providing multiple beneficial effects [62]. One important aspect to consider in HDL research is the variability in HDL-cholesterol levels across studies focused on the same disease. This variability may stem from factors such as the duration of the disease or the presence of comorbidities. Recognizing and addressing these factors could enhance our understanding and further help pave a pathway for more consistent conclusions in future research.

Nevertheless, existing evidence strongly suggests that allergies substantially influence HDL composition and metabolism, which may impact disease progression, infection risk, and cardiovascular health.

## 4. Clinical Evidence

Epidemiological and clinical studies have increasingly illuminated a potential association between allergic diseases and the development of atherosclerosis. There is proof that individuals with chronic allergic conditions, such as atopic dermatitis, asthma, and allergic rhinitis, exhibit an elevated risk of cardiovascular events, including myocardial infarction and stroke [63]. Moreover, allergic inflammation has been associated with worsening of conventional cardiovascular risk factors, including dyslipidemia, hypertension, and obesity [5], raising the possibility that allergic diseases may act more as amplifiers of existing vascular risk than as independent drivers. Notably, the overlapping roles of Th2-mediated immune responses, eosinophils, and mast cells in allergic reactions and vascular inflammation have been identified as potential shared mechanisms underlying this association [64]. However, few studies have directly compared their activity in both contexts within the same cohort or tissue. For example, while mast cell degranulation is well-characterized in late-stage plaques, its role in early plaque formation is less clear, and may differ depending on the type of allergic stimulus or comorbidity. Similarly, the cytokine IL-5 plays a central role in eosinophilic asthma, but its pro-atherogenic role remains inconsistent across studies. This highlights a key limitation in the field: much of the mechanistic overlap is associative rather than demonstrably causal. However, while these associations are statistically significant, their biological plausibility and causality remain only partially understood. For instance, systemic inflammation linked to allergic conditions may accelerate atherogenesis by promoting endothelial dysfunction, lipid oxidation, and leukocyte infiltration in arterial walls. While the exact mechanistic pathways in humans remain to be fully elucidated, epidemiological and clinical biomarkers suggest that allergic inflammation may promote vascular changes consistent with early atherogenesis. Numerous clinical studies have explored the relationship between allergic conditions and cardiovascular diseases (CVDs), particularly ischemic heart disease (IHD), atherosclerosis, and stroke. For instance, a longitudinal cohort study that utilized data from the Korean National Health Insurance Service Health Screening Cohort database (2002–2015) found that patients with asthma exhibit an increased risk of developing IHD, with a hazard ratio of 1.27; notably, this risk is further elevated in individuals experiencing frequent exacerbations. Although asthma was associated with a marginally lower overall risk of stroke, patients prone to exacerbations faced a heightened risk. Furthermore, allergic asthma demonstrated stronger associations with CVDs than non-allergic asthma, underscoring the role of systemic inflammation and immune dysregulation as converging mechanisms [65]. Additionally, the National Health and Examination Survey (NHANES) 2005–2006 and the Wake Forest site of the Multi-Ethnic Study of Atherosclerosis (MESA) cohort have corroborated the association between IgE sensitization to foods and cardiovascular mortality, as established in 2019. The findings suggest that allergic patients, particularly those with severe or allergic forms of asthma, warrant careful monitoring for cardiovascular risk [66]. However, variations in outcome definitions, asthma severity, and treatment exposure across studies complicate interpretation. For example, some analyses relied on self-reported asthma, which may introduce misclassification bias, while others used physician-confirmed diagnoses with clinical outcome tracking. Regarding atopic dermatitis, a meta-analysis of 17 population-based studies found that the severity of atopic dermatitis (AD) is linked to a higher risk of major cardiovascular events, including angina, myocardial infarction (MI), coronary revascularization, heart failure, cardiac arrhythmias, stroke, and cardiovascular death. Specifically, the analysis revealed that AD is associated with an increased risk of several conditions: myocardial infarction (risk ratio [RR] 1.12), stroke (RR 1.10), ischemic stroke (RR 1.17), angina (RR 1.18), and heart failure (RR 1.26) [67]. Importantly, the risk appeared to correlate with disease severity, supporting the hypothesis that chronic systemic inflammation in severe AD may contribute to endothelial dysfunction and atherogenesis. However, many included studies relied on health insurance data or self-reported diagnoses, limiting granularity regarding treatment regimens, disease duration, and inflammatory biomarkers. The impact of allergic rhinitis (AR) on cardiovascular disease (CVD) risk remains a topic of debate. For example, a study analyzing data from 110,207 AR patients in a matched cohort from 1999 to 2012 in South Carolina, USA, found a lower risk of myocardial infarction (MI) (hazard ratio [HR] 0.63), coronary heart disease (HR 0.81), and overall CVD (HR 0.67) among AR patients [68]. In contrast, data from adult participants in the National Health Interview Survey (NHIS), derived from the Integrated Public Use Microdata Series (IPUMS) harmonization project from 1999 to 2018, indicate that physician-diagnosed asthma (both lifetime and past-year prevalence) and allergic rhinitis (AR) in the past year are associated with higher rates of cardiovascular morbidity in the U.S. population [69]. These opposing results may reflect differences in study design, population demographics, AR severity, or the presence of comorbid allergic conditions such as asthma or AD. Overall, the evidence for AR as an independent cardiovascular risk factor remains inconclusive.

Further studies with longitudinal inflammatory profiling and clinical endpoints are needed to establish causality. The implementation of integrated care approaches may prove beneficial in managing these patients.

### 4.1. Implications for Clinical Practice and Future Research: Potential New Therapies Targeting Allergic Inflammation Pathways

Chronic inflammation driven by Th2 cells, which is characteristic of conditions like asthma and atopic dermatitis, may not only lead to allergic symptoms but also contribute to systemic vascular changes that promote the development of atherosclerosis. This connection between immune responses and vascular health suggests the potential for using biologic therapies, created for treating allergic diseases, to modulate cardiovascular inflammation. Among these therapies, agents that target IL-5 (such as mepolizumab and benralizumab) and IL-4/IL-13 (such as dupilumab) have proven effective in reducing eosinophilic and Th2-mediated inflammation, respectively (Table 2). However, while these treatments enhance control over allergic diseases, their impact on vascular inflammation and cardiovascular outcomes has not been extensively studied in dedicated clinical trials.

A recent study utilizing molecular profiling and imaging techniques has revealed that patients with moderate-to-severe atopic dermatitis (AD) show elevated markers of vascular inflammation. These markers include T cell activation, Th2 cytokines, and signaling pathways related to atherosclerosis, found in both skin lesions (affected) and non-lesional intact (unaffected) skin, as well as in the blood. The presence of elevated endothelial microparticles, which indicate damage to blood vessels, were found to be in correlation with both vascular inflammation and the severity of AD. Notably, treatment with dupilumab was associated with downregulation of atherosclerosis-related gene signatures, suggesting a potential systemic vascular benefit [91]. By suppressing cytokine-mediated adhesion molecule expression and immune cell recruitment, these drugs may help mitigate atherogenesis. Yet, this finding remains preliminary and hypothesis-generating, as cardiovascular endpoints were not directly assessed.

Mast cells (MCs) are involved in both the early and advanced stages of plaque formation as well as in allergic diseases and are another promising target. As discussed before, their release of proteases, cytokines, and chemokines contributes to inflammation, neovascularization, and plaque remodeling. Mast cell (MC) stabilizers may help reduce inflammation within atherosclerotic plaques, potentially lowering the risk of acute cardiovascular events, although such approaches remain underexplored in clinical settings. Therapies that inhibit the release of cytokines from mast cells, such as TNF-α and IL-6, may help reduce inflammatory signaling pathways that exacerbate the progression of atherosclerosis [24], but require careful evaluation due to their broad systemic effects. Also, asthma treatments, such as β2-agonists and corticosteroids, can have both positive and negative effects on cardiovascular outcomes depending on dosage, duration, and patient comorbidities. Emerging therapies like omalizumab, an anti-IgE monoclonal antibody, shows potential. However, a cautious approach is required, taking into consideration their potential associations with thromboembolic events, highlighting the need for cardiovascular safety data in allergic populations [92].

An efficient way of treatment could be therapies that address both allergic inflammation and dyslipidemia simultaneously. Statins, known for their lipid-lowering and anti-inflammatory properties, could theoretically complement cytokine-blocking biologics. Although some studies suggest synergy, particularly when statins are combined with IL-1β inhibitors such as canakinumab, for example, Ref. [21], specific evidence in allergic populations is lacking. Future trials should consider stratifying patients by allergic disease status to evaluate whether anti-inflammatory biologics provide added vascular benefit when paired with standard lipid-lowering therapies.

### 4.2. Limitations and Future Perspectives

This review has some limitations, such as diversity in study designs, the exclusion of animal studies, and the possibility of publication bias due to reliance on peer-reviewed literature. Furthermore, the variability of study populations may restrict the applicability of the findings. Future research should include large-scale longitudinal studies to prove causality, investigate the therapeutic possibility of reducing Th2-driven inflammation, and discover specific biomarkers that relate allergic inflammation to atherosclerosis. Further mechanistic investigations are required to determine the precise role of eosinophils, mast cells, and reactive oxygen species in vascular dysfunction.

## 5. Conclusions

In conclusion, the connection between allergic inflammation and atherosclerosis highlights the importance of understanding immune-driven mechanisms in the progression of cardiovascular diseases. Allergic inflammation, which involves Th2 cytokines, eosinophil activity, mast cell degranulation, and changes in lipid metabolism, contributes to endothelial dysfunction, chronic vascular inflammation, and plaque progression. While individual allergic conditions like asthma and atopic dermatitis have been linked to increased cardiovascular risk, the strength and consistency of this association vary by disease type and severity. Importantly, current data are largely observational, and mechanistic insights in humans remain limited, often extrapolated from preclinical models. Emerging therapies, such as cytokine inhibitors, mast cell stabilizers, and biologics targeting specific pathways, present promising options for addressing both allergic inflammation and cardiovascular complications. Future research should prioritize longitudinal cohort studies that integrate inflammatory biomarkers, allergy phenotypes, and vascular imaging to clarify causal pathways. Clinical trials assessing the vascular effects of anti-cytokine therapies in high-risk allergic populations are also warranted. By bridging gaps in knowledge, we can advance personalized treatment strategies that address the dual effects of allergic inflammation and atherosclerosis, ultimately improving patient outcomes and reducing the burden of chronic inflammatory diseases on global health.

## Figures and Tables

**Table 1:** The roles of Th2 cells, mast cells, and eosinophils in atherosclerosis and their mechanism of action.

Type of Cell	Mechanism of Action	Effect
Th2 Cells	Secrete IL-4, IL-5, and IL-13, promoting a Th2-skewed inflammatory response. Induce expression of adhesion molecules (VCAM-1, ICAM-1) on endothelial cells, enhancing immune cell recruitment. Suppress Th1 responses and modulate macrophage polarization [21].	Increase endothelial dysfunction, enhances monocyte infiltration into plaques, and promote macrophage differentiation. Contribute to chronic vascular inflammation, foam cell formation, and plaque instability [22].
Mast Cells	Degranulate in response to allergens, releasing histamine, TNF-α, IL-6, VEGF, tryptase, chymase, and leukotrienes.Release histamine, cytokines (e.g., TNF-α, IL-6), growth factors (e.g., VEGF), proteases (e.g., tryptase, chymase) and other pro-inflammatory mediators upon activation induce smooth muscle contraction and vascular remodeling [23].	Enhance the permeability of blood vessels, promoting the adhesion of leukocytes (white blood cells) to the endothelial cells lining the blood vessels. Facilitate the infiltration of LDL particles and inflammatory cells in the arterial intima. Promote extracellular matrix degradation, neovascularization, and intraplaque hemorrhage, contributing to plaque rupture [24].
Eosinophils	Secrete major basic protein (MBP), eosinophil cationic protein (ECP), and reactive oxygen species (ROS). Produce lipid-oxidizing enzymes and cytokines (e.g., IL-5, IL-13) [25].	Enhance oxidative stress and endothelial dysfunction, promoting atherosclerotic plaque formation. Induce endothelial damage and smooth muscle cell activation. Contribute to plaque instability through pro-inflammatory cytokine release [26].

**Table 2:** Potential new therapies targeting the allergic inflammatory pathway.

Therapy Type	Target/Mechanism	Examples	Therapeutic Goals
Monoclonal Antibodies	IgE neutralization	Omalizumab	Prevent mast cell and basophil activation [70]
	IL-4/IL-13 inhibition	Dupilumab	Reduce Th2 cytokine signaling, IgE production [71]
	IL-5/IL-5R blockade	Mepolizumab, Benralizumab	Decrease eosinophil survival and activation [72,73]
	TSLP inhibition	Tezepelumab	Block epithelial cytokine that initiates allergic response [74]
	IL-33/ST2 inhibition	Astegolimab (experimental)	Suppress type 2 innate immunity [75]
	IL-25 blockade	Under development	Reduce type 2 inflammation from epithelial signals [76]
	Siglec-8 targeting	Antolimab	Induce eosinophil apoptosis [77]
Small Molecule Inhibitors	JAK inhibitors (block cytokine signaling)	Upadacitinib, Abrocitinib	Broad suppression of inflammatory signaling [78,79]
	CRTh2 antagonists	Fevipiprant (investigational)	Inhibit Th2 cell migration and activation [80]
	Syk inhibitors	Fostamatinib (investigational)	Block FcεRI-mediated mast cell activation [81]
Immunotherapy Enhancements	Peptide-based immunotherapy	Allergen peptides (e.g., Cat-PAD)	Induce tolerance without risk of anaphylaxis [82]
	DNA/RNA allergen vaccines	Under clinical development	Modulate immune response toward tolerance [83]
	Adjuvant-enhanced AIT	TLR agonists, CpG motifs	Promote regulatory T cell response [84]
Microbiome-Based Therapies	Probiotics/Prebiotics	*Lactobacillus* spp., *Bifidobacterium* spp.	Modulate gut–skin–lung immune responses [85]
	Fecal Microbiota Transplant (FMT)	Experimental	Restore immune balance through microbiota reset [86]
Cell-Based Therapies	Regulatory T cell (Treg) therapy	Autologous/engineered Tregs	Suppress allergic inflammation [87]
	CAR-Tregs (Chimeric Antigen Receptor Tregs)	Preclinical	Allergen-specific immune tolerance [88]
Gene/RNA Therapies	siRNA/microRNA targeting cytokines	Under research	Suppress key inflammatory mediators [89]
	CRISPR-based editing	Preclinical	Disrupt genes involved in IgE or Th2 responses [90]
